# Frailty Related to the Exposure to Particulate Matter and Ozone: The Korean Frailty and Aging Cohort Study

**DOI:** 10.3390/ijerph182211796

**Published:** 2021-11-10

**Authors:** Jinyoung Shin, Jaekyung Choi

**Affiliations:** Department of Family Medicine, Research Institute on Healthy Aging, Konkuk University Medical Center, Konkuk University School of Medicine, Seoul 05030, Korea; jyshin@kuh.ac.kr

**Keywords:** frail elderly, air pollution, particulate matter, ozone

## Abstract

This study aims to identify the association between the concentration of particulate matter <2.5 μm (PM_2.5_), <10 μm (PM_10_), and ozone (O_3_) and frailty. The Korean Frailty Scale (KFS, 0–6 points) assessing physical, psychological, and social frailty, was applied to 2912 community-dwelling older adults between April 2016 and December 2017. Daily average concentrations of PM_2.5_, PM_10_, and O_3_ (2015–2017) were obtained and matched with the residential areas. The frailty risk associated with exposure to PM_2.5_, PM_10_, and O_3_ was evaluated using multiple logistic regression after adjusting for age, sex, BMI, lifestyle, socioeconomic status, and comorbidity. Participants were categorized into robust (0 points, 28.7%), pre-frail (1–2 points, 50.1%), and frail (≥3 points, 21.2%) groups. Each 1 μg/m^3^ increase of PM_2.5_ and PM_10_ increased the odds ratios (ORs) and 95% confidence intervals (CIs) of the frail group compared to the robust group: 1.055 (1.002, 1.112) and 1.095 (1.060, 1.131), and the pre-frail group: 1.053 (1.017, 1.090) and 1.062 (1.037, 1.087), respectively. Each 1-ppb increase of O_3_ increased the OR (95% CI) of the frail group: 1.041 (1.023, 1.059) and the pre-frail group: 1.005 (0.985, 1.025). PM_2.5_, PM_10_, and O_3_ may be associated dose-dependently with the frailty.

## 1. Introduction

Frailty is caused by aging-related functional declines. It confers a high risk of adverse health outcomes, such as hospitalization, difficulty performing activities of daily living (ADLs), poor quality of life, and early mortality [1,2,3,4]. The adverse health outcomes of frailty can occur even without any comorbid illness and can worsen the quality of life of the elderly [5,6]. Therefore, effective strategies to prevent or manage frailty in an aging population must be established to reduce the burden of health care for geriatric society [5]. 

In the air quality standards defined by the World Health Organization (WHO) in 2005, particulate matter (PM) and ozone (O_3_) were the most harmful pollutants [7]. Research studies focusing on the links between air pollution and the health status of older adults have suggested that air pollution reduces life expectancy, increases respiratory mortality and chronic disease, and impairs mental health [8,9,10,11,12]. Air pollution exposure may affect the frailty of older adults, who are particularly susceptible to its adverse effects by accelerating a failure of homeostatic mechanisms [13]. Nevertheless, the association between frailty and air pollution is heterogeneous because few studies have examined that relationship. In 26,026 older adults (≥65 years old) residing in New Taipei City in 2020, high levels of particulate matter ≤2.5 μm (PM_2.5_) exposure for 12 months increased the risk of pre-frail and frail using the Fried Frailty Phenotype (FFP) questionnaire [14]. However, exposure to particulate matter was generally not associated with physical function among 1782 Dutch older adults (mean age 75.5 ± 8.5) in roughly the same time frame [15]. Therefore, the association between air pollution and frailty may be affected by the differences in race, lifestyle, type, concentration, or exposure duration of the pollutants, or the frailty measurements [16,17]. On the other hand, the effects of O_3_ on frailty are currently unknown. O_3_, a secondary pollutant caused by photochemical reactions, produces an inflammatory response, oxidative stress, and tissue injury [17,18]. Acute exposure to O_3_ decreased the life expectancy of a frail population [19], but the effect of long-term exposure to O_3_ on frailty has not been explored.

This study investigated the association between PM_2.5_, PM ≤ 10 μm (PM_10_), O_3_, and frailty, measured by a comprehensive assessment tool using Korean Frailty and Aging Cohort Study (KFACS) data.

## 2. Materials and Methods

### 2.1. Study Participants

The KFACS is a nationwide longitudinal study undertaken in 10 urban, rural, and suburban communities every two years since 2016 [20]. They recruited participants using quota sampling stratified by age (70–74, 75–79, and 80–84 years with a ratio of 6:5:4, respectively) and sex (male and female) in diverse settings (local senior welfare centers, community health centers, apartments, housing complexes, and outpatient clinics) to minimize selection bias [20]. In this study, we enrolled 3014 community-dwelling older adults who could visit the participating clinical sites between April 2016 and December 2017. However, 102 subjects who had moved within one year were excluded from the analysis. Ultimately, we analyzed 2912 participants (96.6% of the initial enrollments).

This study was approved by the Institutional Review Board (IRB) of the Clinical Research Ethics Committee of a Medical Center (IRB File No. KUH 2021-07-026). The requirement for informed consent was waived because we used only de-identified and previously collected data.

### 2.2. Frailty Measurements

We assessed frailty status using the Korean Frailty Scale (KFS), a comprehensive and multi-dimensional frailty scale validated in community-dwelling older adults [21]. The KFS consists of six questionnaires that assess physical frailty (weight loss and self-assessment of health status), psychological frailty (fatigue and loss of energy), and social frailty (social network and support), which was confirmed as effective for predicting falls, mortality, and functional decline by measuring instrumental activities of daily living [21]. Weight loss was defined as the unintentional loss of ≥4.5 kg or 5% of body weight compared with the preceding year. Self-assessment of health status was evaluated by asking patients how they perceived their health. The response of “poor” was scored one point, and “not poor” was zero points [22]. Fatigue was measured by asking respondents how much time during the past four weeks they felt tired, with the responses “all the time” or “most of the time” being scored one point [23]. We evaluated lack of energy by asking, “Have you dropped many of your activities and interests?” with the participant answering either “yes” or “no” [24]. As for social frailty, the social network was defined as a social structure unit composed of the individual’s social ties and the ties among them by asking, “Do you sometimes meet or talk to your friend(s)?” [25,26]. We defined a poor social network as one in which people meets or talks with their friends less than once a month. Social support also arises from personal relationships in the context of both formal support groups and informal helping relations [25]. We measured levels of social support by asking, “Do you have as much contact as you would like with someone you feel close to, someone whom you can trust and confide in?” [27]. If no or little time was spent with someone, we defined that as poor social support. Frailty status in the KFS was categorized in either three groups (robust [0 points], pre-frail [1–2 points], and frail [≥3 points]) or two groups (non-frail [0–2 points] and frail [≥3 points]).

We additionally compared the associations between four commonly used scales, the FFP, the Frailty Instrument (FI), the Korean Frailty Index (KFI), and the Study of Osteoporotic Fracture (SOF) frailty index, because frailty prevalence is heterogeneous depending on the definition or components of frailty [28]. The Fried Frailty Phenotype (FFP) questionnaire was validated in the Cardiovascular Health Study [2] and included unintentional weight loss, exhaustion, low physical activity measured by the International Physical Activity Questionnaire (IPAQ) short form, weakness (handgrip strength <26 kg for men and <18 kg for women), and slowness (walking speed <1.0 m/s over 4 m). The sum of each component scored as one point, and the classification groups were frail (3–5 points) and non-frail (0–2) [28]. The FI is a simple questionnaire with three components—grip strength, self-reported exhaustion, and social isolation—to predict disability, institutionalization, and mortality for Korean older adults in the National Long-term Care Service [29]. Scoring two or more points in the FI results is classified as a frail group. The Korean Frailty Index (KFI) is an eight-item questionnaire: hospital admission in the preceding year, self-assessment health status, polypharmacy (≥four prescriptions), recent weight loss with loosening of clothing, depressive mood, incontinence, auditory and/or visual disturbance, and timed get-up-and-go test (≥10 s) [22]. Each component scored one point, and the cutoff value for the frail group was ≥5. The modified SOF frailty index includes unintentional weight loss of 5% more over one year, the actual time needed to stand up five times within 60 s and feeling reduced energy [30]. Each component ranged from 0 to 1, and the sum of the scores was classified as non-frail (0–1) or frail (2–3).

Physical performance was measured with the short physical performance battery (SPPB) and timed up and go (TUG) test. The SPPB included the ability to stand for up to 10 s in three ways (feet positioned together side-by-side, semi-tandem, and tandem), time to complete a 4 m walk, and time to rise from a chair times. Each item in the SPPB was scored from zero to four point, with the total score ranging from zero (worst) to 12 (best) points. We defined low physical performance as less than eight total points [31,32]. The TUG test was measured as the time needed to stand up from a chair, walk 3 m, and then return to the starting seated position; cut-offs of 11.8 s for men and 12.5 s for women identified poor physical performance [31]. Appendicular skeletal muscle (ASM) mass and skeletal muscle index as ASM/height^2^ were measured by dual-energy X-ray absorptiometry (Lunar; GE Healthcare, Madison, WI and Hologic DXA; Hologic Inc., Bedford, MA, USA) according to the recommendation of the European Working Group on Sarcopenia in Older People (EWGSOP) in 2018 [33]. Low muscle quantity was defined as ASM < 20 kg in men and <15 kg in women and SMI as <7.0 kg/m^2^ in men and <6.0 kg/m^2^ in women [34].

### 2.3. Air Pollutant Variables

The average hourly concentrations of PM_2.5_, PM_10_, and O_3_ were measured at 268 nationwide surveillance stations in 2015–2017, covering most residential areas of South Korea except for mountains and the green areas. The air pollution surveillance stations and residential areas were matched when the five-digit codes were the same. The monthly concentrations of PM_2.5_, PM_10_, and O_3_ from January 2015 to enrolled time were calculated as the individual levels. PM_10_ and PM_2.5_ were measured using a β-ray attenuation system (PM-711D; Dongil Greensys Co., Ltd., Seoul, Korea). O_3_ was measured using ultraviolet photometry (202; Total Engineering Co., Ltd., Yongin-si, Gyeonggi-do, Korea) according to the Korean Air Pollutants Emission Service standard operating procedure of the National Institute of Environmental Research (Incheon, Korea).

### 2.4. Other Variables

Smoking status was classified as never smoker, former smoker, or current smoker. Alcohol intake was classified by drinking frequency: never or less than once per week, and more than once per week. Physical activity was evaluated using the IPAQ and divided into active and inactive groups according to energy expenditure, <20% of the average for participants in this study (<494.65 kcal for men and <283.50 kcal for women). Participants were divided into groups according to marital status (married/with a partner, and divorced/widowed/unmarried), education level (<9, ≥9 years), current employment (yes, no), and household income (<1,000,000 won/month, ≥1,000,000 won/month). The residential areas were divided into urban, suburban, or rural. Body mass index (BMI) was calculated as weight divided by height squared (kg/m^2^). The Charlson’s Comorbidity Index was used to indicate each participant’s overall health status [35].

### 2.5. Statistical Analyses

Continuous variables for demographics are expressed as median values and minimum and maximum values, and categorical variables are expressed as numbers and percentages. We compared the characteristics of the study subjects among the robust, pre-frail, and frail groups using the Kruskal–Wallis test and the chi-square test. Because the level of air pollutants, meteorological data, and frailty scales were not normally distributed according to the Shapiro–Wilk test, we evaluated the relationship between the average annual concentrations of air pollutants, meteorological data, and the frailty scales using Spearman’s correlation analysis (Supplementary Tables S1 and S2).

Logistic regression analyses were performed to evaluate the odds ratios (ORs) and 95% confidence intervals (CIs) of the frail and pre-frail groups per 1 μg/m^3^ increase of PM_2.5_ and PM_10_, per 1 ppb increase of O_3_, and per 1 standard deviation (SD) increase, and the quartiles of pollutants after adjusting for age, sex, smoking, alcohol intake, physical activity, BMI, education, income, marital status, residential area, and comorbidity; this overall assessment was Model 1. In addition to Model 1, Model 2 considered the meteorological data, including temperature, rainfall, humidity, wind speed, and sunshine time during the study period for which the participants were recruited (during 2016 and 2017). Model 3 also considered PM_2.5_, PM_10_, or O_3_ levels because the particulate matter and O_3_ are correlated.

We assessed the associations between the KFS components (weight loss, self-assessment of health status, fatigue, lack of energy, social network, and support) and each 1 μg/m^3^ or 1 ppb, and each 1 SD increase of the pollutants after adjusting for the confounding factors. We also compared the effects of PM_2.5_, PM_10_, and O_3_ on frailty classified on other frailty scales. Stratified analyses to investigate possible effect modifications by age (75 years old), sex, smoking (never, ever), education (divided by 9 years: higher, lower), physical activity (divided by 20% of the average for the participants: active, inactive), and income status (1,000,000 won/month: higher, lower) were performed for each 1 μg/m^3^ increase of PM_10_ compared to the non-frail group. *P*-interaction values were calculated by adding an interaction term (each risk factor and pollutant). A two-tailed *p*-value of < 0.05 was considered statistically significant. All analyses were performed using IBM SPSS Statistics for Windows, version 24.0 (IBM Corp., Armonk, NY, USA). 

## 3. Results

### 3.1. Air Pollutants Concentration and Meteorological Data

The levels of air pollutants and the meteorological parameter data, including the daily average temperature, humidity, wind speed, sunshine time, and yearly mean rainfall, are presented in Table 1. 

### 3.2. Demographic Characteristics of the Study Population

Among the 2912 subjects, 835 (28.7%) were identified as being in the robust group, 1460 (50.1%) in the pre-frail group, and 617 (21.2%) in the frail group (Table 2). The frail group had greater proportions of older adults and females, had never smoked, and was physically inactive compared to those in the robust group. However, the ratio of current smoking was high. Also, the frail group was less likely to drink, had lower education and income levels, was more likely to reside in rural areas, was divorced/widowed/unmarried, and had more comorbid diseases than the other groups. However, there was no significant difference in current employment status.

### 3.3. Association between Particular Matter, Ozone and Frailty 

As shown in Table 3, we found that increased concentrations of PM_2.5_, PM_10_, and O_3_ were associated with an elevated risk of being frail and pre-frail compared to the robust group. The OR (CI) of PM_2.5_ per 1 μg/m^3^ increase in frailty was 1.055 (1.002, 1.112) in Model 1, but the association between PM_2.5_ and frailty disappeared after additionally adjusting for meteorological data. The OR of PM_10_ per 1 μg/m^3^ increase in frailty was 1.095 (1.060, 1.131) in Model 1, 1.106 (1.056, 1.158) in Model 2, and 1.188 (1.093, 1.290) in Model 3. The OR of O_3_ per 1 ppb increase in frailty was 1.041 (1.023, 1.059) in Model 1 and 1.093 (1.031, 1.160) in Model 2, while there was no significant association in Model 3. The association between frailty and PM_2.5_, PM_10_, and O_3_ also increased per every 1 SD increase. Similar associations between pre-frailty and PM_2.5_, PM_10_, and O_3_ were confirmed. In the comparisons of the frail group and non-frail group, frailty was associated with the increase of PM_2.5_, PM_10_, and O_3_. 

**Figure 1:**
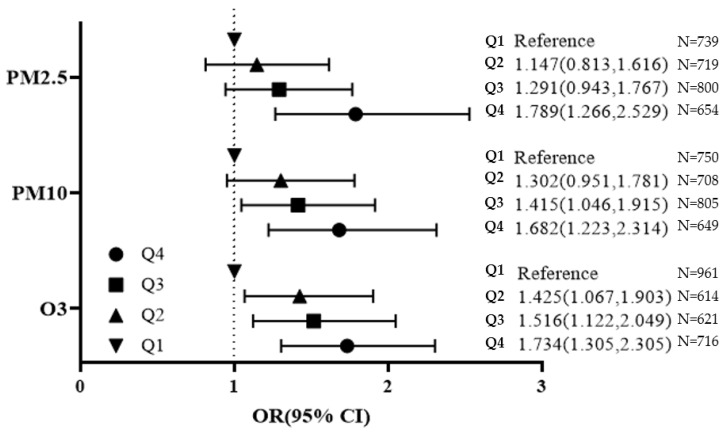
Frailty risk by the Korean Frailty Scale (KFS) according to quartiles of PM_2.5_, PM_10_, and O_3_.

The increase of quartiles in PM_2.5_, PM_10_, and O_3_ showed an increased OR for frailty compared to the lowest level of pollutants in Figure 1. The ORs for frail, prefrail, and physical performance were demonstrated according to quartiles of PM_2.5_, PM_10_, and O_3_ (Supplementary Tables S3 and S4).

Odds ratios (95% confidence intervals) of frailty are presented. Adjusting for age, sex, smoking, alcohol intake, physical activity, body mass index, education, income, marital status, residential area, comorbidity, and meteorological data.

Using the KFS, the increase of PM_2.5_ was associated with poor health status and lower social networks (Table 4**)**. The increase of PM_10_ was associated with poor health status, fatigue, lack of energy, and lower social network. The increase of O_3_ was associated with weight loss, fatigue, and lack of energy.

Adjusted for age, sex, smoking, alcohol consumption, physical activity, body mass index, education, income, marital status, residence, comorbidity, and meteorological data

Using the FFP, a 10 μg/m^3^ increase of PM_10_ was associated with frailty, and a 10 ppb increase of O_3_ was associated with frailty using the SOF frailty index (Table 5). However, there were no associations between PM_2.5_, PM_10_, O_3_, and frailty measured by the FI and KFI.

**Figure 2:**
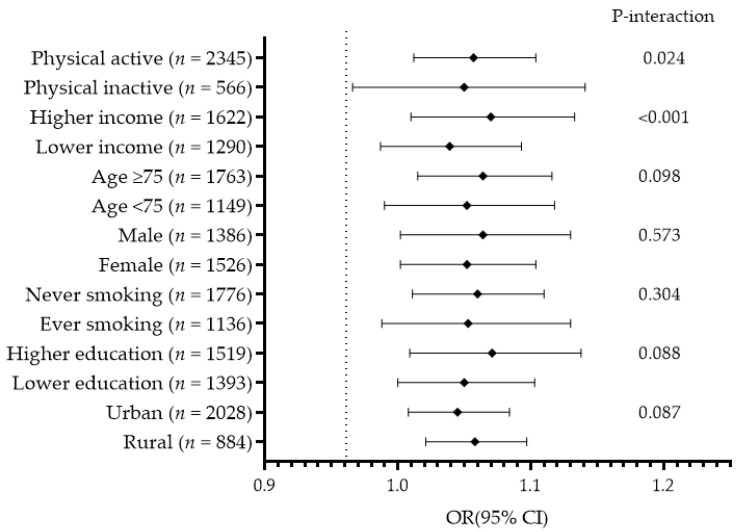
The association between exposure to PM_10_ and frailty according to participants’ characteristics. Adjusted for age, sex, smoking, alcohol intake, physical activity, body mass index, education, income, marital status, residential area, comorbidity, and meteorological data except the standard variable.

The associations between PM_10_ and frailty according to age, sex, lifestyle, and socioeconomic status are presented in Figure 2. In the subgroup analysis, physically active participants had higher income, were aged ≥ 75, never smoked, and had higher education had increased frailty risk. However, physical activity and income status significantly interacted in the associations between PM_2.5_, PM_10_, O_3_, and frailty (*P*-interaction < 0.05).

## 4. Discussion

In the present cross-sectional study on Korean older adults, we identified the associations between more than a year of exposure to PM_2.5_, PM_10_, and O_3_ and frailty considering health behaviors, socioeconomic factors, and comorbidities. We found these associations not only in the frail group but also in the pre-frail group, and the effects of PM_2.5_, PM_10_, and O_3_ were greater in the frail group than in the pre-frail group. In particular, a strong association between PM_10_ and frailty was found independently after adjusting for the various confounding factors. The effects of PM_2.5_, PM_10_, and O_3_ on frailty were presented as physical frailty, psychological frailty, and social frailty. The previous evidence for a link between air pollution and frailty is limited and comes from studies of patients with cardiovascular disease in susceptible populations [36,37,38] or cognitive impairment as a frailty factor in community-dwelling older adults [39]. To date, only the effects of PM_2.5_ on frailty have been reported, without assessment of PM_10_ or O_3_ [14]. This study adds to the existing research on air pollutants and frailty demonstrating links to PM_2.5_, PM_10_, and O_3_ on the various frailty scales and physical performance in the general population of older adults, with the adjustment of various confounding factors using multi-dimensional frailty scales.

Air pollution is known to have an adverse health effect by inducing inflammation, oxidative stress, metabolic disorders, and genetic and epigenetic alterations. Exposure to pollutants can potentiate the age-related decline and deterioration of functional properties at the cellular, tissue, and organ level [40]. A frail person with a low physical performance may be vulnerable to environmental pollution due to a decline in their biologic capacity, resulting in compromised functions [5,13,41]. Furthermore, increased air pollution may have reduced the chances of going out or participating in gatherings, increasing social frailty, affecting physical and psychological frailty as an essential key element in maintaining healthy behaviors [42].

We found that the association of pollutants and frailty was heterogeneous according to the frailty index or measurement of physical performance. Most frailty indices are quantitative measures elucidating the accumulation of deficits. Despite attempts to standardize it, frailty prevalence as measured by various frailty indices differs depending on the components [28]. In one study, frailty prevalence was 21.1% by the KFS and 17.7% by the FFP in the same participants [21]. Although frailty associations with pollutants have been identified throughout the KFS index, such as self-assessed health status, fatigue, lack of energy, and social network, these subjective components may be easily affected by the ambient environment [43]. Physical performance as indicated by hand-grip strength was associated with the air pollution in six low- and middle-income countries [44]. However, low physical performance and muscle quantity were heterogeneously associated with PM_2.5_, PM_10_, and O_3_ in this population_._ Therefore, studies using the components of the frailty tools or the measurement of physical performance keep in mind that the results may vary depending on those tools. As a first study for the frailty and the concentrations of PM_2.5_, PM_10_, and O_3_, further study is needed to evaluate these associations according to participants’ characteristics or morbid conditions. In a study of older adults in Taiwan, the increase of PM_2.5_ was associated with frailty measured by FFP [14]. We also assessed the association between PM_2.5_ and frailty measured by FFP in this study. However, we found no association between PM_2.5_ and frailty using FFP, although the PM_2.5_ concentration in KFACS was higher than in the Taiwan study (median value: 17.7 μg/m^3^ vs. 25.0 μg/m^3^). This discrepancy might be due to the additional effects of BMI, physical activity, or meteorological data that were not considered in the Taiwan study [14]. We found a positive association between PM_2.5_ and frailty in Model 1; therefore, the characteristics and collinearity of other related factors must be considered in the association between pollutants and frailty. 

The effect of pollutants on frailty was greater in men, although the prevalence of frailty was higher in women than in men. Although the ORs for frailty according to sex in this study were not significantly different, it can be carefully suggested that even if the frailty prevalence is higher in women, women live longer and the mortality rate of men is higher [45,46], because of the greater impact of pollutants on men than women. Of course, there are many other factors involved besides air pollution. 

The effect of physical activity and income status on the association between PM_10_ and frailty has been evaluated. Especially, a distinct effect of PM_10_ was found in physically active and higher-income groups. Other studies to date, as well as our own, have shown that the prevalence of frailty increases in physically inactive and lower-income groups [3,28]. Because there is an increased risk of inhaling air pollution while exercising or using transportation, active groups can be more affected by air pollution than inactive groups [47]. In the Hong Kong Elderly Health Service Cohort, assessed in 2020, there was no interaction between physical activity and PM_2.5_, while the risk of cardiovascular and respiratory mortality was decreased where 50% of the population was exposed to similar levels of PM_2.5_ as in our study [48]. Most of the research so far has said that the beneficial mortality effects of habitual physical activity outweigh the detrimental effects of long-term exposure to air pollution [47,48].

Higher incomes lead to increased access to medical care and health-promoting behaviors [49]. Rising income triggers efforts to reduce air pollution sources or the associations between air pollution and income status suggested the inverted U-shape [50]. In the U.S., the increased income of $1000 and 1 μg/m^3^ decrease PM_2.5_ related to human longevity [49]. In contrast, 7358 Chinese older adults with higher income had more difficulty in ADL and cognitive dysfunction increasing air pollution index than those with lower income [51]. We found a positive association between the concentration of PM_10_ and income level in our study (correlation coefficient: 0.083, *p* < 0.001; data not shown in the tables). Historical and contemporary evidence suggests that rapid economic growth immediately causes adverse health outcomes; KFACS participants with high incomes might have not yet benefitted by living in cleaner environments, farther away from hazardous materials [51].

This study has several limitations. First, it was not possible to establish the causality between frailty and exposure to PM_2.5_, PM_10_, and O_3_, and the effect of pollutants on the transitions among the frailty states (robust, pre-frail, and frail) is not evaluated because this is a cross-sectional study. Frailty is a dynamic process, characterized by frequent transitions between frailty states over time [52]. Therefore, further investigation was needed to analyze the proper assignment of the exposure on the change of frailty using polynomial regression models with adequate control of confounding factors. Second, we need to consider biases arising from the exposure assignment to exclude subjects due to unmatching the location of air pollution surveillance stations with their residential area. Third, because the KFACS was conducted on community-dwelling older adults who can move freely, we cannot generalize the association between frailty and air pollutants for older adults living in nursing homes or other specialized care facilities. We could not obtain the concentration of individual exposure data on traffic-related or indoor air pollution. Therefore, further study is needed to identify those associations in the more vulnerable older adults. 

## 5. Conclusions

Exposure to PM_2.5_, PM_10_, and O_3_ was associated with physical, psychological, and social frailty and low physical performance in Korean older adults aged ≥ 70. Physical activity and income status may significantly affect on the associations between PM_2.5_, PM_10_, O_3_, and frailty. It is necessary to carefully interpret these associations based on accumulated research between air pollution and frailty because of heterogeneous associations with various frailty scales and physical performance or muscle quantity. Nevertheless, understanding these associations is necessary for developing frailty-specific prevention and restorative interventions.

## Figures and Tables

**Table 1:** Distribution of annual concentration of particulate matter and ozone and meteorological data.

Variables	Mean	SD	IQR	Percentiles				
				Minimum	25th	50th	75th	Maximum
Air Pollutants								
PM_2.5_, μg/m^3^	25.3	3.2	3.6	19.0	23.5	25.0	27.1	38.2
PM_10_, μg/m^3^	48.0	4.9	5.4	36.8	46.7	48.7	52.1	66.3
O_3_, ppb	28.3	6.0	7.0	19.0	24.0	27.0	31.0	45.0
Meteorological data								
Temperature, °C	13.4	1.3	1.0	11.7	12.6	13.0	13.6	17.0
Rainfall, mm	1131.5	257.4	241.5	766.7	991.7	1023.4	1233.2	1734.6
Humidity, %	65.3	4.3	10.1	59.3	59.3	65.7	69.4	70.2
Wind speed, m/s	1.7	0.6	0.4	0.9	1.4	1.6	1.8	3.2
Sunshine, hours	6.4	0.5	0.5	5.0	6.2	6.6	6.7	6.8

Particulate matter < 2.5 μm (PM_2.5_), Particulate matter < 10 μm (PM_10_), and Ozone (O_3_) in the research places were measured between 2015 and 2017. Daily average temperature, humidity, wind speed, sunshine time, and yearly mean rainfall were represented between 2016–2017. Meteorological data were obtained in the open data portal of Korea Meteorological Administration; https://data.kma.go.kr/resources/html/en/aowdp.html (accessed on: 25 June 2021).

**Table 2:** Baseline characteristics of study population (*n* = 2912).

Variables	Robust	Pre-Frail	Frail	*p*-Values
Number, N (%)	835 (28.7)	1460 (50.1)	617 (21.2)	
Age, years	75.4 ± 3.7	76.0 ± 3.9	76.8 ± 3.8	<0.001
Sex				<0.001
Male	480 (57.5)	679 (46.5)	227 (36.8)	
Female	355 (42.5)	781 (53.5)	390 (63.2)	
Smoking				<0.001
Never	453 (54.3)	898 (61.5)	425 (68.9)	
Former	335 (40.1)	482 (33.0)	149 (24.1)	
Current	47 (5.7)	80 (5.5)	43 (7.0)	
Alcohol intake				<0.001
Never/Less than one time per week	515 (61.7)	1055 (72.3)	478 (77.5)	
More than one time per week	320 (38.3)	405 (27.7)	139 (22.5)	
Physical activity, kcal/week				<0.001
Active	753 (90.2)	1190 (81.5)	402 (65.2)	
Inactive	82 (9.8)	270 (18.5)	215 (34.8)	
Education, years				<0.001
<9	284 (34.0)	696 (47.7)	413 (66.9)	
≥9	551 (66.0)	764 (52.3)	204 (33.1)	
Marital status				<0.001
Married/with partner	633 (75.8)	974 (66.7)	376 (60.9)	
Divorced/widowed/unmarried	202 (24.2)	486 (33.3)	241 (39.1)	
Household income, won/month				<0.001
<1,000,000	286 (34.3)	644 (44.1)	360 (58.3)	
≥1,000,000	549 (65.7)	816 (55.9)	257 (41.7)	
Residential area				<0.001
Urban	263 (31.5)	423 (29.0)	122 (19.8)	
Suburban	357 (42.8)	600 (41.1)	263 (42.6)	
Rural	215 (25.7)	437 (29.9)	232 (37.6)	
Current employment				0.072
Yes	200 (24.0)	408 (27.9)	152 (24.6)	
No	635 (76.0)	1052 (72.1)	465 (75.4)	
Body mass index, kg/m^2^	24.5 ± 2.8	24.5 ± 3.0	24.1 ± 3.4	0.013
Carlson’s comorbidity index	3.17 ± 0.37	3.24 ± 0.43	3.29 ± 0.42	<0.001

Data were shown as N (%) or mean ± standard deviation.

**Table 3:** Odds ratios (ORs) and 95% confidence intervals (CIs) of the frailty according to the increases of PM_2.5_, PM_10_, and O_3_.

Pollutants		ORs (95% CIs)		
		Model 1	Model 2	Model 3
Robust vs. Frail				
PM_2.5_	1 μg/m^3^	1.055 (1.002,1.112)	1.065 (0.974,1.165)	0.81 (0.702,0.962)
PM_10_	1 μg/m^3^	1.095 (1.060,1.131)	1.106 (1.056,1.158)	1.188 (1.093,1.290)
O_3_	1 ppb	1.041 (1.023,1.059)	1.093 (1.031,1.160)	1.021 (0.953,1.094)
PM_2.5_	1 SD	1.326 (1.154,1.524)	1.209 (0.923,1.583)	0.554 (0.345,0.890)
PM_10_	1 SD	1.574 (1.337,1.853)	1.655 (1.314,2.083)	2.364 (1.563,3.575)
O_3_	1 SD	1.271 (1.146,1.410)	1.709 (1.202,2.431)	1.133 (0.751,1.710)
Robust vs. pre-frail				
PM_2.5_	1 μg/m^3^	1.053 (1.017,1.090)	1.030 (0.970,1.094)	0.802 (0.717,0.898)
PM_10_	1 μg/m^3^	1.062 (1.037,1.087)	1.072 (1.040,1.105)	1.168 (1.104,1.236)
O_3_	1 ppb	1.005 (0.985,1.025)	1.057 (1.015,1.101)	0.999 (0.953,1.047)
PM_2.5_	1 SD	1.167 (1.052,1.294)	1.093 (0.912,1.310)	0.516 (0.368,0.724)
PM_10_	1 SD	1.348 (1.198,1.518)	1.414 (1.216,1.645)	2.175 (1.642,2.882)
O_3_	1 SD	1.029 (0.912,1.161))	1.395 (1.093,1.781)	0.992 (0.748,1.315)
Non-frail vs. frail				
PM_2.5_	1 μg/m^3^	1.051 (1.014,1.090)	1.039 (0.968,1.116)	0.905 (0.797,1.028)
PM_10_	1 μg/m^3^	1.052 (1.024,1.080)	1.058 (1.019,1.098)	1.098 (1.027,1.173)
O_3_	1 ppb	1.024 (1.002,1.048)	1.051 (1.001,1.102)	1.017 (0.962,1.074)
PM_2.5_	1 SD	1.162 (1.043,1.294)	1.123 (0.908,1.388)	0.742 (0.506,1.087)
PM_10_	1 SD	1.287 (1.128,1.468)	1.324 (1.100,1.593)	1.593 (1.142,2.223)
O_3_	1 SD	1.155 (1.010,1.322)	1.344 (1.007,1.793)	1.104 (0.793,1.536)

Model 1: age, sex, smoking, alcohol consumption, physical activity, body mass index, education, income, marital status, residence, and comorbidity; Model 2: Model 1 + meteorological data; Model 3: Model 2 + other PMs and ozone.

**Table 4:** Association between particular matter, ozone, and KFS components (*n* = 2912).

Pollutant	Weight Loss	Poor Health Status	Fatigue	Lack of Energy	Lower Social Network	Lower Social Support
PM_2.5_	0.929 (0.841,1.026)	1.077 (1.012,1.145)	0.998 (0.933,1.067)	0.984 (0.927,1.044)	1.098 (1.023,1.178)	1.013 (0.932,1.102)
PM_10_	1.013 (0.966,1.063)	1.045 (1.014,1.078)	1.037 (1.004,1.072)	1.037 (1.007,1.067)	1.079 (1.041,1.119)	1.024 (0.972,1.078)
O_3_	1.117 (1.034,1.205)	0.997 (0.957,1.038)	1.075 (1.026,1.126)	1.064 (1.022,1.108)	0.977 (0.928,1.028)	0.922 (0.868,0.978)

Per 1 μg/m^3^ or 1 ppb increase.

**Table 5:** Association between particular matter, ozone, and several frailty scales.

Pollutant	FFP *	FI	KFI	SOF Frailty Index
PM_2.5_	1.544 (0.602,3.960)	1.711 (0.671,1.359)	0.661 (0.224,1.950)	0.784 (0.202,3.041)
PM_10_	1.689 (1.066,2.676)	1.516 (0.950,2.418)	0.923 (0.544,1.567)	1.253 (0.625,2.510)
O_3_	1.888 (0.971,3.670)	0.969 (0.475,1.977)	0.895 (0.436,1.836)	4.367 (1.451,13.139)

Per 10 μg/m^3^ or 10 ppb increase; adjusted for age, sex, smoking, alcohol consumption, physical activity, body mass index, education, income, marital status, residence, comorbidity, and meteorological data; FFP: Fried frailty phenotype scale, FI: frailty instrument, KFI: Korean Frailty Index, SOF frailty index: Study of Osteoporotic Fracture Frailty Index; * physical activity was excluded as a confounding factor of this analysis.

## Data Availability

The data presented in this study are available on request from the corresponding author.

## Supplementary Materials

The following are available online at https://www.mdpi.com/article/10.3390/ijerph182211796/s1, Table S1: Correlation between air pollutants and meteorological data, Table S2: Correlation between air pollutants and frailty scales, Table S3. Polynomial logistic regression analysis for the association PM_2.5_, PM_10_, O_3_ and frailty by KFS, Table S4. Odds ratios (95% confidence intervals) of low physical performance or muscle quantity according to quartiles of PM_2.5_, PM_10_, and O_3_.
