# Supplementary material for: Frailty Related to the Exposure to Particulate Matter and Ozone: The Korean Frailty and Aging Cohort Study
Source: Int J Environ Res Public Health. 2021 Nov 10;18(22):11796. doi: 10.3390/ijerph182211796 (PMC8623935; doi:10.3390/ijerph182211796)
Supplement: Supplementary file 1 [file ijerph-18-11796-s001.zip › ijerph-1446333-supplementary.pdf]

Table S1. Correlation between air pollutants and meteorological data

	$PM_{2.5}$	PM_{10}	O_3	Temperature	Rainfall	Humidity	Wind speed	Sunshine
$PM_{2.5}$	1	0.761*	-0.202*	-0.276*	0.310*	-0.002	-0.089*	0.156*
PM_{10}		1	-0.109*	-0.236*	0.009	0.030	-0.089*	0.167*
O_3			1	0.514*	-0.176*	0.566*	0.528*	0.737*
Temperature				1	0.035	0.204*	0.810*	0.712*
Rainfall					1	0.183*	-0.007	0.015
Humidity						1	0.161*	-0.561*
Wind speed							1	-0.774*
Sunshine								1

* $P < 0.001$, ** $P < 0.05$: two-tailed.

Particulate matter <10 μm (PM_{10}), particulate matter < 2.5 μm ($PM_{2.5}$), and ozone (O_3) were measured from 2015 to 2017. Korea Meteorological Administration, Seoul, Korea https://data.kma.go.kr/resources/html/en/aowdp.html

Table S2. Correlation between air pollutants, frailty scales, and physical measurements

	$PM_{2.5}$	PM_{10}	O_3	KFS†	FFP†	FI†	KFI†	SOF†	SPPB‡	TUG†	ASM‡	SMI‡
$PM_{2.5}$	1	0.758*	-0.195*	0.049**	0.045**	0.037**	0.069*	0.044**	0.037**	-0.080*	0.065*	0.133*
PM_{10}		1	-0.108*	0.074*	0.113*	0.079*	0.072*	0.046**	0.105*	-0.066*	0.079*	0.166*
O_3			1	0.081*	0.052*	0.024	0.010	0.055**	-0.030	-0.015	-0.129*	-0.197*
KFS				1	0.463*	0.452*	0.548*	0.532*	-0.157*	0.244*	-0.206*	-0.174*
FFP					1	0.703*	0.439*	0.386*	-0.145*	0.366*	-0.238*	-0.176*
FI						1	0.369*	0.302*	-0.134*	0.254*	-0.235*	-0.159*
KFI							1	0.412*	-0.207*	0.463*	-0.161*	-0.119*
SOF								1	-0.166*	0.228*	-0.229*	-0.222*
SPPB									1	-0.236*	0.145*	0.168*
TUG										1	-0.136*	-0.119*
ASM											1	0.906*
SMI												1

* $P < 0.001$, ** $P < 0.05$: two-tailed.

KFS: Korean frailty scale, FFP: Fried frailty phenotype scale, FI: frailty instrument, KFI: Korean frailty index, SOF frailty index: Study of osteoporotic fracture frailty index, SPPB: Short physical performance battery, TUG test: Timed up and go test, ASM: appendicular skeletal, and SMI: Skeletal muscle index

Particulate matter <10 μm (PM_{10}), particulate matter < 2.5 μm ($PM_{2.5}$), and ozone (O_3) were measured from 2015 to 2017.

†Lower score means better health status. ‡Higher score means better health status.

Table S3. Polynomial logistic regression analysis for the association $PM_{2.5}$, PM_{10}, O_3 and frailty by KFS

	Model 1		Model 2		Model 3	
	Frail	Prefrail	Frail	Prefrail	Frail	Prefrail
$PM_{2.5}$						
Q1	1	1	1	1	1	1
Q2	1.221(0.776,1.920)	1.317(0.870,1.995)	1.208(0.698,2.091)	1.025(0.560,1.876)	0.630(0.281,1.413)	0.309(0.128,0.743)
Q3	1.756(1.074,2.869)	1.444(0.916,2.276)	1.259(0.654,2.423)	1.481(0.910,2.411)	1.227(0.724,2.079)	0.764(0.423,1.380)
Q4	2.306(1.466,3.627)	1.417(0.952,2.109)	1.894(0.990,3.623)	1.742(0.951,3.191)	1.284(0.619,2.662)	0.785(0.360,1.712)
PM_{10}						
Q1	1	1	1	1	1	1
Q2	1.485(0.946,2.333)	1.133(0.762,1.685)	1.974(1.146,3.403)	1.418(0.862,2.331)	2.424(1.308,4.491)	1.215(0.560,2.633)
Q3	1.761(1.023,3.031)	1.231(0.756,2.005)	2.972(1.482,5.961)	1.460(0.919,2.318)	4.091(1.670,10.019)	1.390(0.821,2.354)
Q4	3.047(1.953,4.755)	1.399(0.932,2.099)	3.325(1.894,5.837)	1.930(1.044,3.568)	4.771(1.981,11.487)	1.707(0.774,3.766)
O_3						
Q1	1	1	1	1	1	1
Q2	0.965(0.641,1.452)	0.539(0.334,0.872)	0.602(0.248,1.465)	1.071(0.505,2.272)	0.386(0.142,1.052)	1.189(0.525,2.693)
Q3	1.288(0.844,1.966)	1.228(0.780,1.932)	0.783(0.321,1.913)	1.090(0.496,2.395)	0.687(0.299,1.579)	1.249(0.522,2.991)
Q4	1.679(1.046,2.694)	1.555(0.920,2.631)	1.309(0.630,2.718)	1.385(0.721,2.662)	0.756(0.280,2.041)	1.377(0.669,2.837)

Model 1: age, sex, smoking, alcohol consumption, physical activity, body mass index, education, income, marital status, residence, and comorbidity
Model 2: Model 1 + meteorological data
Model 3: Model 2 + other PMs and ozone

Table S4. Odds ratios (95% confidence intervals) of low physical performance or muscle quantity according to quartiles of $PM_{2.5}$, PM_{10}, and O_3

Pollutants	N	Poor SPPB	Poor TUG test	Low ASM	Low SMI
$PM_{2.5}$					
Q_1	739	1	1	1	1
Q_2	719	1.222(0.826,1.807)	0.810(0.497,1.321)	1.359(0.886,2.083)	1.577(1.041,2.330)
Q_3	800	1.572(1.001,2.468)	0.806(0.444,1.461)	0.950(0.648,1.393)	0.94090.653,1.351)
Q_4	654	1.868(1.145,3.048)	0.902(0.479,1.699)	0.364(0.253,0.525)	0.534(0.374,0.763)
PM_{10}					
Q_1	750	1	1	1	1
Q_2	708	1.014(0.694,1.482)	0.686(0.321,1.192)	1.294(0.811,2.066)	1.106(0.720,1.699)
Q_3	805	1.088(0.732,1.616)	0.735(0.450,1.201)	0.747(0.518,1.0780	0.646(0.454,0.919)
Q_4	649	1.910(1.159,3.150)	0.901(0.563,1.441)	0.282(0.198,0.402)	0.272(0.192,0.387)

O_3						
Q_1	961	1	1	1	1	Adjusted by age, sex, smoking, alcohol consumption, physical activity, body mass index, education, income, marital status, residence, and comorbidity
Q_2	614	1.096(0.563,2.134)	0.936(0.524,1.671)	0.307(0.202,0.466)	0.194(0.123,0.306)	
Q_3	621	1.560(0.900,2.702)	1.127(0.545,2.332)	1.709(1.111,2.630)	0.865(0.571,1.309)	
Q_4	716	1.750(0.903,3.390)	1.911(0.969,3.767)	3.348(2.301,4.872)	2.433(1.695,3.492)	
